# Activity of Caudate Nucleus Neurons in a Visual Fixation Paradigm in Behaving Cats

**DOI:** 10.1371/journal.pone.0142526

**Published:** 2015-11-06

**Authors:** Tamás Nagypál, Péter Gombkötő, Balázs Barkóczi, György Benedek, Attila Nagy

**Affiliations:** 1 Department of Physiology, Faculty of Medicine, University of Szeged, Szeged, Hungary; 2 Center for Molecular and Behavioral Neuroscience Rutgers University, Newark, New Jersey, United States of America; McGill University, CANADA

## Abstract

Beside its motor functions, the caudate nucleus (CN), the main input structure of the basal ganglia, is also sensitive to various sensory modalities. The goal of the present study was to investigate the effects of visual stimulation on the CN by using a behaving, head-restrained, eye movement-controlled feline model developed recently for this purpose. Extracellular multielectrode recordings were made from the CN of two cats in a visual fixation paradigm applying static and dynamic stimuli. The recorded neurons were classified in three groups according to their electrophysiological properties: phasically active (PAN), tonically active (TAN) and high-firing (HFN) neurons. The response characteristics were investigated according to this classification. The PAN and TAN neurons were sensitive primarily to static stimuli, while the HFN neurons responded primarily to changes in the visual environment i.e. to optic flow and the offset of the stimuli. The HFNs were the most sensitive to visual stimulation; their responses were stronger than those of the PANs and TANs. The majority of the recorded units were insensitive to the direction of the optic flow, regardless of group, but a small number of direction-sensitive neurons were also found. Our results demonstrate that both the static and the dynamic components of the visual information are represented in the CN. Furthermore, these results provide the first piece of evidence on optic flow processing in the CN, which, in more general terms, indicates the possible role of this structure in dynamic visual information processing.

## Introduction

The basal ganglia, which are components of cortical and subcortical loops in the mammalian brain [1] are strongly involved in sensorimotor functions. It is assumed that for the eliciting of the normal motor behavior in response to sensory information from the environment the basal ganglia are essential. Thus it comes as no surprise that the CN neurons are sensitive to various modalities of visual stimulation [2–9]. Beside the characterization of classical visual receptive field properties of the CN neurons [5, 10] their responsiveness to extended visual stimuli was also investigated [11, 12]. They were markedly sensitive to very low spatial and high temporal frequencies and exhibited narrow temporal and spatial frequency tuning. These characteristics suggest that these neurons can be involved in the processing of dynamic visual information [12–14]. An especially relevant dynamic aspect of the incoming visual information is the apparent motion of the environment during self-motions, that is, optic flow. Logical as this question might be, so far it has not been investigated if it is possible to activate CN neurons directly by an optic flow stimulus, while a positive answer could throw new light on the CN as an important structure of dynamic visual processing during self-motion. For that reason, the first question we ask in this study is whether such a direct activation is possible.

Neuroanatomical studies revealed several distinct neuron groups in the CN. Of these, medium spiny neurons, GABAergic interneurons and cholinergic interneurons are the most often observed [15]. Similarly, electrophysiological studies in rodents and primates described three large groups based on the recorded neurons' electrophysiological properties: phasically active (PAN), high-firing (HFN) and tonically-firing (TFN) neurons [16–20]. It is known from these studies that there is a strong correspondence between the anatomical and electrophysiological clusters. PANs correspond to the medium spiny projection neurons, HFNs to the GABAergic interneurons and the TFNs to the cholinergic interneurons [21–28]. The second question raised by the present study is whether the same or a similar classification is possible in the feline brain.

To answer the questions raised above, we used behaving, head-restrained eye movement-controlled cats trained for chronic visual electrophysiological recordings [29] in a behavioral paradigm. We categorized the neurons of the feline CN according their electrophysiological properties in different functional clusters and analyzed their responsiveness to static as well optic flow visual stimuli.

## Materials and Methods

Experiments were carried out on two adult feline domestic cats (2.6 kg and 3.25 kg). Cats were trained for the applied visual fixation task. All experimental procedures were carried out to minimize the number and the discomfort of the animals involved and followed the European Communities Council Directive of 24 November 1986 (86 609 EEC) and the National Institutes of Health guidelines for the care and use of animals for experimental procedures. The experimental protocol was accepted and approved by the Ethics Committee for Animal Research of the University of Szeged (No: I-74-24/2012).

### Animal Preparation and Surgery

The animals were initially anesthetized with ketamine hydrochloride (Calypsol (Gedeon Richter^®^), 30 mg/kg i.m). To reduce salivation and bronchial secretion, a subcutaneous injection of 0.2 ml 0.1% atropine sulphate was administered preoperatively. A cannula was inserted in the femoral vein and after intubation of the trachea the animals were placed in a stereotaxic headholder. All wounds and pressure points were treated regularly with local anesthetic (1%, procaine hydrochloride). Throughout the surgery the anesthesia was maintained with 1.5% halothane in a 2:1 mixture of N_2_O and oxygen. The depth of anesthesia during the surgery was monitored by continuously checking the end-tidal halothane concentration and heart rate (electrocardiogram). The end-tidal halothane concentration, MAC values and the peak expired CO_2_ concentrations were monitored with a capnometer (CapnomacUltima, Datex-Ohmeda, ICN). The O_2_ saturation of the capillary blood was monitored by pulse oxymetry. The minimum alveolar anesthetic concentration (MAC) values calculated from the end-tidal halothane readings were kept in the range recommended by Villeneuve and Casanova [30]. The peak expired CO_2_ concentration was kept within the range 3.8–4.2% by adjusting the respiratory rate or volume. The body temperature of the animal was maintained at 37°C by a computer-controlled, warm-water heating blanket. Craniotomy was performed with a dental drill to allow a vertical approach to the target structures. The dura mater was preserved, and the skull hole was covered with a 4% solution of 37°C agar dissolved in Ringer’s solution. Then a reclosable plastic recording chamber (Ultem 1000, internal diameter = 22.5mm) was mounted on the skull. Following this, the eight electrodes were implanted in the brain with the help of an adjustable microdrive system (a modified Harper-McGinty microdrive for the first animal (see McKown and Schadt, [31]) and a modified Korshunov microdrive for the second animal [32]). In order to control the eye movements, a scleral search coil was implanted into the right eye [33]. To restrain the head of the animal during the experiments, a stainless steel headholder was cemented on the skull. All surgical procedures were carried out under aseptic conditions. Before the surgical procedure, antibiotic prophylaxis was applied (1000 mg ceftriaxon, i.m., Rocephin 500 mg (Roche^®^)). The first five postoperative days, 50 mg/kg i.m. antibiotic was provided. Nalbuphin and non-steroidal anti-inflammatory drugs were administered until the seventh postoperative day.

### Brief Description of the Behavioral Training of the Animals

When the cat got accustomed to the laboratory environment, it was carefully clothed into a canvas harness. First, we lifted the animal manually only a few centimeters from the floor. When the clothed animal got used to being suspended, it was gradually introduced to the experimental stand. The experimental stand is a cubical structure with each side open, in which the suspension harness is fastened at two points in by a rope pulley block. The head of the suspended cat was fixed to the stereotaxic frame by the implanted steel headholder with two stainless steel bars. The stereotaxic frame was placed within an electromagnetic field, which is generated by metal coils, installed into the wall of the stand. The animal was trained to perform fixation during recordings of neuronal activities to different kind of visual stimulation. If a trial of the task was completed without the breaking the fixation the animal received mashed food reward through a plastic tube, dosed by a computer-driven hydraulic pump installed outside the magnetic field. The behavioral training and later the recording sessions lasted approximately 2 hours per a day, four to five times during a week. The weight of the animals was checked regularly and was kept at least 90% of the initial value. The detailed description of the implantation and the behavioral training of the animal can be found in our recent methodological paper [29].

### Behavioral Paradigm and Visual Stimulation

For visual stimulation and the projection of the fixation point a standard 17- inch CRT monitor (refresh rate: 100 Hz) was placed in front of the animal, at a distance of 57 cm. During the recordings the effect of the eye movements on the neuronal activity has to be controlled for, so the cats were trained to perform visual fixation during the visual stimulation. For this, a fixation point was projected on the center of the CRT monitor. The size of the fixation point was 0.8° in diameter. The animal had to look at the fixation point and keep its gaze within a square fixation acceptance window during the trials of the visual fixation task. The size of the acceptance window was 5°. The stimulation task was initiated by the animal, by keeping fixation in the acceptance window for 500 ms. Two sorts of stimuli were applied: first a static random dot pattern, which was followed by an optic flow stimulus. The size of the stimulation screen was 40.5 ° by 30.5 °. The size of each dot in both the static and the dynamic stimulus was 0.1° in diameter. The speed of dot movement in the optic flow increased from 0 to 7°/sec toward the periphery of the stimulation screen. The stimuli were generated using a custom made script prepared in the Psychtoolbox of Matlab®.

**Fig 1:**
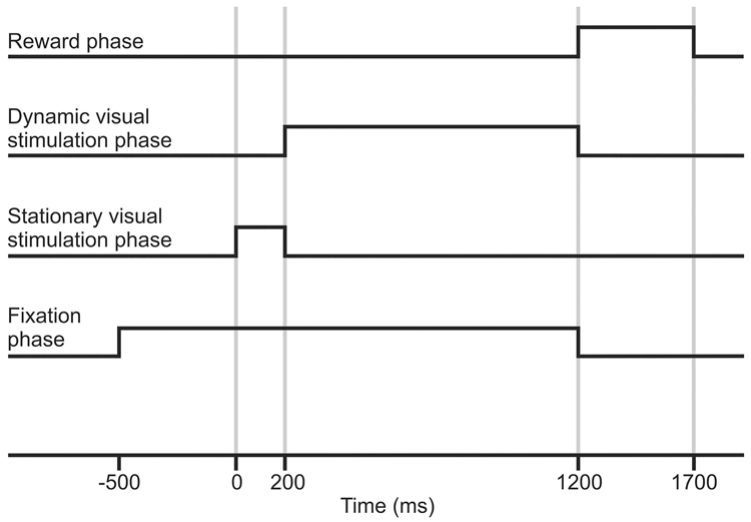
Schematic representation of the phases of the behavioral paradigm. At the beginning of the trial the cat has to direct its gaze to a green fixation point projected in the center of a CRT monitor (refresh rate 100 Hz). Then, the animal has to maintain fixation within an acceptance window for 500 ms (fixation phase). If the fixation phase is successfully accomplished, a static random dot pattern appears (at 0 ms), and it remains on the screen for 200–500 ms (static stimulation phase). After this, the dots of same static pattern start to move coherently in a radial center-in or center-out direction (optic flow, dynamic stimulation phase, 1000 ms). As can be seen in the figure, the animal has to hold fixation throughout the stimulation phases. If it manages to do so, the trial is accepted, and the animal is rewarded with a few drops of mashed cat food (reward phase, 500 ms).

Fig 1 is a schematic representation of a single trial of the applied behavioral visual fixation paradigm. To minimize the influence of eye movements on the neuronal activity, the animal had to hold fixation during the trials. A trial was immediately aborted if the animal broke fixation, and in this case no reward was given. Any single trial consisted of the following phases:

1. Fixation phase: a green fixation point appeared in the center of the stimulation screen. In this phase the cat had to direct its gaze to the fixation point and keep fixation in a pre-determined fixation window (see above) for 500 ms. The accomplishment of this initiated the next phase.
2. Static stimulation phase (200–500 ms): immediately after the successful completion of the fixation phase, a static random dot pattern appeared in the visual field.
3. Dynamic stimulation phase (1000 ms): the dots of the static pattern began to move radially (optic flow). The dots moved either toward the periphery of the screen (center-out optic flow) or toward the center of the screen (center-in optic flow).
4. Reward phase (500 ms post stimulation) and intertrial interval (4000–10000 ms): If the cat managed to maintain fixation throughout all the stimulation phases of a single trial, a few drops of pulpy food reward was given and we considered the trial as a correct one. This phase was longer, so that the cat could eat the reward without muscle activity interfering with the recordings of the next trial. The actual recordings started when the cats reached a stable 80% efficiency in fixation maintenance in the training sessions. The recordings took place in a dark laboratory room (background luminance: 0.5 cd/m^2^). The luminance of the stimulus was 5 cd/m^2^ (Mavolux 5032C, GOSSEN Foto- und Lichtmesstechnik GmbH, Germany). The computer-controlled trials (either with center-out or center-in optic flow) were presented in a random order. A recording session was considered successful (and the data included in the analysis) if the cat performed at least 30 correct trials to center-out and also to center-in optic flow stimuli.

### Recording and Data Analysis

Extracellular multielectrode recordings were carried out with eight implanted parylene isolated platinum-iridium wire-electrodes (diameter: 25 μm) from the first cat and with eight implanted formvar insulated Nickel-Chrome wire-electrodes (diameter: 50 μm) from the CN of the second cat. The position of the guiding tube (diameter = 0.65 mm), which contained the wire-electrodes during implantation, was anterior 13 and lateral 5.5 mm according to the Horsley-Clarke coordinates. The position of the single wire-electrodes ranged between anterior 12.5–14 mm, lateral 4.5–6 mm at stereotaxic depths between 9 and 13.5 mm. At the end of the experiments, the first animal was deeply anesthetized with pentobarbital (200 mg/kg i.v.) and perfused transcardially with 4% paraformaldehyde solution. The brain was removed and cut into coronal sections of 40 μm, and the sections were stained with DAPI (4',6-diamidino-2-phenylindole, Sigma-Aldrich Co., USA). Recording sites were localized on the basis of the marks of the electrode penetrations.

Amplified neuronal activities were band-pass filtered (300 to 5000 Hz). The raw data were first processed by NeuroScope, NDManager, KlustaKwik and then broken down into single unit signals by the use of Klusters [34, 35] under manual control.

**Fig 2:**
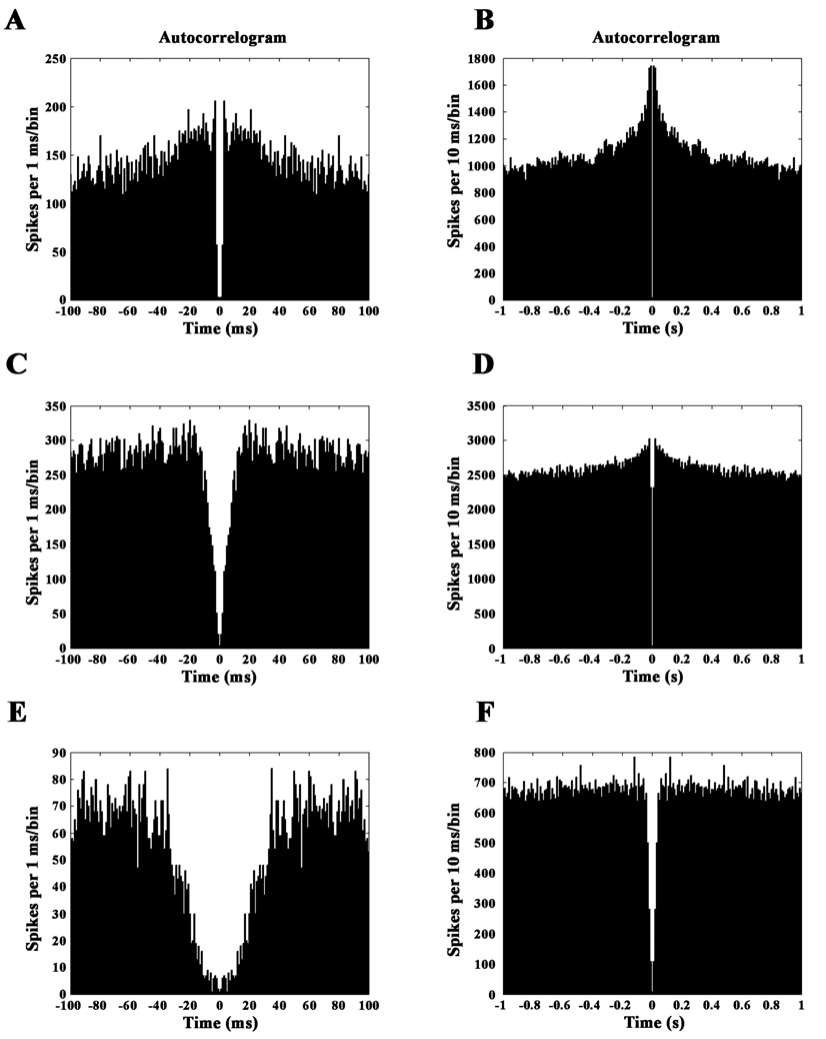
Autocorrelograms of the CN neurons. Neurons were classified on the basis of the shape of their autocorrelograms (at 100 ms and 1000 ms time resolutions), propISI_>2sec_ and the background discharge rate in three big groups (PAN, HFN, TAN). Neurons belonging to each group have characteristic autocorrelogram. PANs are usually characterized by peaky autocorrelogram (A,B). HFNs have autocorrelograms with a blunt peak (C,D) and TANs are characterized by a deep gap in the autocorrelogram (E,F).

We intended to classify the CN neurons in functional groups by their electrophysiological properties. It is known from rat and primate studies that spontaneous activity, the proportion of the summed values over 2 seconds divided by the total session time in the interspike intervals (propISI_>2sec_), and the shape of the autocorrelogram at different time resolutions (100ms, 1000 ms) can be used to classify the CN neurons in different groups [16, 18]. The above mentioned electrophysiological parameters were calculated for each recorded CN unit. The spontaneous discharge rate of each neuron was calculated based on the last 3000 ms period of the intertrial intervals. In this way, the recorded neurons could be classified in three groups (Fig 2): PANs are characterized by peaky autocorrelogram and ISI values over 2 seconds. The propISI_>2sec_ was usually higher than 0.5, and the spontaneous discharge rate was low, in most cases under 3 spikes/sec. The HFNs have autocorrelograms with a blunt peak, the propISI_>2sec_ is lower than 0.5, and the spontaneous discharge rate is higher than 5 spikes/sec. Finally, TANs are characterized by a deep gap in the autocorrelogram, the propISI_>2sec_ is lower than 0.5, and the spontaneous discharge rate is between 2 and 12 spikes/sec.

Firing rates during the different phases of the behavioral paradigm (static, dynamic, reward) were compared to the background activity, which was measured in the last 3000 ms period of the intertrial interval, with the Mann-Whitney rank-sum test. We considered the neuronal activity as response if the stimulated activity in a particular phase or phases of the paradigm was significantly different (p<0.05) from the background activity. Similarly, the comparison between discharge rate of each optic-flow sensitive single neuron in response to center-in and center-out optic flow stimuli was performed using Mann-Whitney test. The neuron was considered to be direction sensitive if the difference between the responses to center-in and center-out stimulation was significant (p<0.05). We also calculated the net firing rate of each unit by subtracting their background activity from the summed firing of the neurons. The background activities and the net firing rates of different CN neuron subpopulations (PAN, HFN and TFN) in different epochs of the paradigm were compared by one-way analysis of variance (ANOVA). In the case of significant variance (p<0.05) Tukey's HSD analysis was performed to find the significantly different CN neuron subpopulation. All statistical analyses were performed in Matlab® (MathWorks Inc., Natick, MA).

Eye movements were monitored and recorded with a search coil system (DNI Instruments, Newark, DE, USA) at a sampling rate of 250 Hz, and these were processed by a custom software written under Matlab®.

The recording of the eye movements, stimulus presentation, reward delivery, and data collection were also coordinated by a custom software via 16 channels National Instrument Card DAQ®.

## Results

Altogether 346 units were recorded from the CN. The primary aim of the present study was to describe the visual response characteristics of the CN neurons. Based on their electrophysiological properties (see in methods), we could group the recorded CN neurons in three major clusters: PANs (221 neurons), HFNs (88 neurons) and TANs (28 neurons). Our secondary aim was to compare the response characteristics of these neuronal functional groups. Table 1 summarizes the responsivenes of the different CN neuron clusters. Because of the very low spontaneous discharge rate (below 1 spike/second), we excluded 135 PANs from the further analysis. Further nine CN neurons were excluded from the analysis, because it was not possible to classify them as belonging to any of the three major clusters. After all the exclusions, responses from 202 neurons were analyzed. The responsive CN neurons showed mainly increased firing rate, while decreased activity was also found during the different phases of the visual behavioral paradigm. Significant changes in the activity of the CN neurons were recorded not only during the actual visual stimulation. In line with earlier studies [36], reward-related neuronal responses were also recorded shortly before and during the reward period (provided that the task was successfully completed).

**Table 1:** The number of responsive CN neurons by the different phases of the applied paradigm.

	Static	Optic flow		Stimulus off	Reward
		center-out	center-in		
Phasically active neurons					
increased activity	26	17	16	1	27
decreased activity	24	2	6	0	6
					
High-firing neurons					
increased activity	18	16	20	7	30
decreased activity	18	9	8	1	0
					
Tonically active neurons					
increased activity	11	3	5	0	6
decreased activity	2	1	1	1	0

In the following we are describing the stimulus-related response characteristics of the functional clusters.

### Response Characteristics of the Phasically Active Neurons

**Fig 3:**
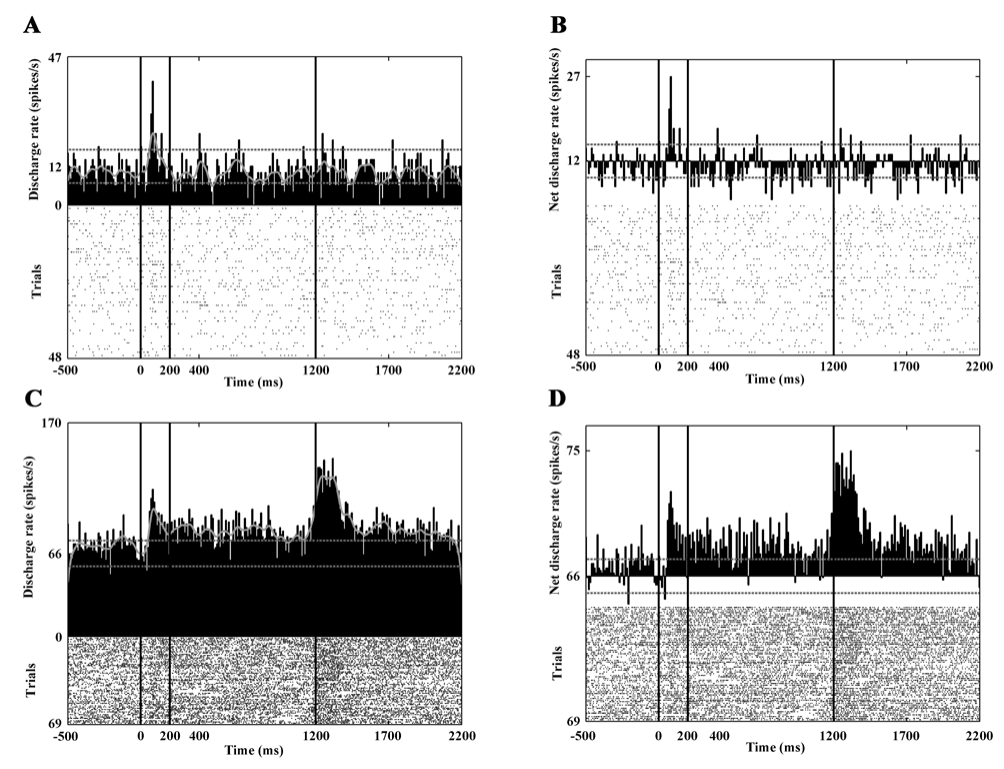
Response characteristics of the CN neurons to static visual stimulation. Each panel of the figure contains a PSTH (top) and a raster plot (bottom) to represent the activity of a neuron. Panels A and B show the activity of a PAN, panels C and D show the firing pattern of a HFN. A and C show the gross activities, B and D show the net activity change after the subtraction of the background activity. The vertical black lines denote the boundaries between the different phases of the paradigm: fixation phase (-500 to 0 ms), static visual stimulation (0 to 200 ms), dynamic visual stimulation (200 to 1200 ms), reward phase (1200 to 1700 ms). Note the phasic response of the PAN to static stimulation (A,B). The activity pattern of the presented HFN (C,D) is more complex: increased activity can be observed not only to random dot patterns, but also to optic flow and during the reward phase. The abscissa denotes the time in milliseconds. The ordinate denotes the number of successful trials and the activity of the neuron. The lowest number is the number of the successful trials, the next is the average discharge during the whole recording and the highest number is the stimulated activity (Hz). The continuous grey curve is a smoothed curve of the activity, and the dashed grey lines indicate ± 2 SD of the average background discharge rate in the whole recording.

After the exclusion of the neurons that exhibited spontaneous activity lower than 1 spike/s, the response characteristics of 86 PANs were analyzed. The mean spontaneous discharge rate was 2.93 spikes/sec (SD: ±2.18). Overall, the visual responses of the PANs were moderate or weak (Table 2). During static visual stimulation, significant activity change was observed in 50 neurons. In 26 cases this meant a significant increase, and in 24 cases a significant decrease was seen. Fig 3A and 3B show the responses of a PAN CN neuron to static visual stimulation.

**Table 2:** The net discharge rates (spikes/sec) of the PANs, HFNs and TANs by the different phases of the applied paradigm.

	Phasically active neurons					
	N	Mean	Median	SD	Min	Max
Background activity	86	2.93	2.34	2.18	0.97	8.88
Static						
increased activity	26	1.91	0.72	3.34	0.23	16.51
decreased activity	24	0.55	0.44	0.52	0.06	1.43
Optic flow:center-out						
increased activity	17	0.94	0.87	0.48	0.38	1.92
decreased activity	2	0.37	0.37	0.13	0.28	0.46
Optic flow:center-in						
increased activity	16	1.34	1.04	0.90	0.41	3.60
decreased activity	6	0.88	0.88	0.33	0.50	1.32
Reward						
increased activity	27	3.11	2.14	2.65	0.53	10.09
decreased activity	6	0.38	0.18	0.47	0.12	1.32
	High-firing neurons					
	N	Mean	Median	SD	Min	Max
Background activity	88	14.45	12.85	6.81	4.67	37.91
Static						
increased activity	18	8.51	7.92	4.51	1.80	19.52
decreased activity	18	3.18	1.88	3.75	0.45	15.91
Optic flow:center-out						
increased activity	16	5.34	4.47	3.80	0.99	14.48
decreased activity	9	1.13	1.12	0.57	0.29	1.77
Optic flow:center-in						
increased activity	20	4.56	3.51	3.63	0.30	12.84
decreased activity	8	0.79	0.85	0.38	0.24	1.33
Reward						
increased activity	30	9.57	7.06	7.05	1.82	32.65
decreased activity	0	-	-	-	-	-
	Tonically active neurons					
	N	Mean	Median	SD	Min	Max
Background activity	28	5.24	5.81	2.37	1.26	10.11
Static						
increased activity	11	1.69	1.20	1.49	0.43	4.83
decreased activity	2	0.23	0.23	0.23	0.06	0.39
Optic flow:center-out						
increased activity	3	1.24	1.36	0.23	0.98	1.38
decreased activity	1	0.21	-	-	-	-
Optic flow:center-in						
increased activity	5	0.97	0.98	0.59	0.32	1.85
decreased activity	1	0.81	-	-	-	-
Reward						
increased activity	6	2.89	1.86	3.79	0.24	9.91
decreased activity	0	-	-	-	-	-

Increased activities are given as net values after the subtraction of the background activity from the gross activity. Decreased activities are given as absolute values of the net changes.

**Fig 4:**
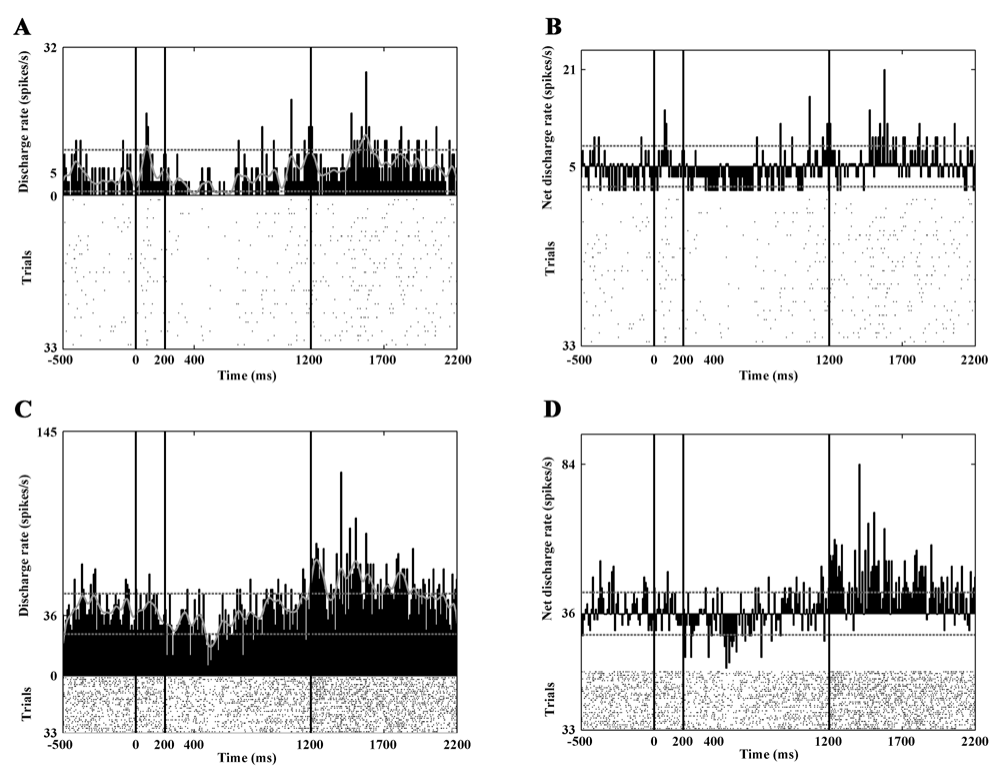
Decreased responses to optic flow stimulation. Panels A and B show the activity of a PAN, panels C and D show the firing pattern of a HFN. Note the marked activity decrement during optic flow stimulation. The conventions are the same as in Fig 3.

Twenty-nine neurons showed activity change during dynamic visual stimulation. During the center-out optic flow 17 of them, while during the center-in optic flow 16 of them showed increased activity. In eight cases decreased activity was seen in response to this type of stimulation. The peristimulus time histograms (PSTHs) in Fig 4A and 4B show such a response.

**Fig 5:**
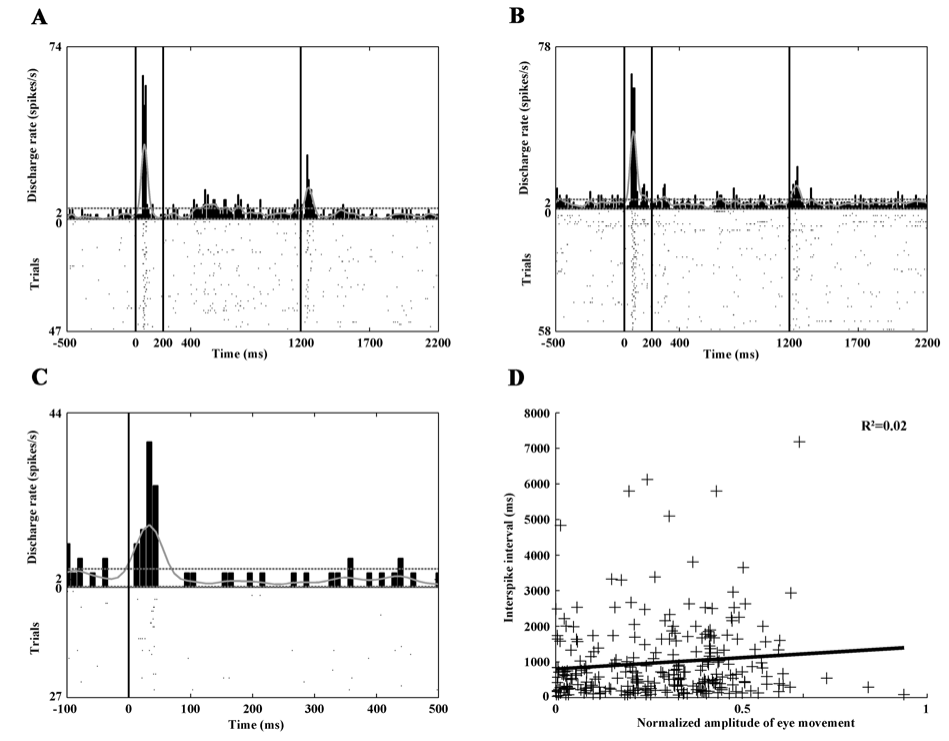
Stimulation-related response of a PAN. The PSTHs in panels A and B show the activity of the neuron in response to stimulation. A: response to center-out optic flow. B: response to center-in optic flow. The response to random dot patterns (A,B), to center-out optic flow (A) but not to center-in (B) and the beginning of the reward phase are readily observable (A,B). The conventions are the same as in Fig 3. In order to decide whether the increased activity at the beginning of the reward phase is purely reward-related or related to stimulus offset, the aborted trials (where the cat had broken the fixation during visual stimulation and therefore got no reward) were also analyzed (C). Panel C is aligned to the time of the breaking of the fixation, which corresponded to the offset of the stimulus because the trial was immediately aborted upon fixation breaking. Note the PSTH peak, which indicates responsiveness to the offset of the stimulus. Furthermore, in order to control for the effects of saccadic activity, the correlation between the interspike intervals and the normalized amplitude of the eye movements recorded during the experiment was computed (D). The linear regression analysis clearly shows the lack of connection (ρ^2^: 0.02, p>0.05). This means the activity is of no saccadic origin.

In the reward phase 27 neurons showed significantly increased activity and six of them decreased their discharge rate. The question arises whether this activity is purely reward-related and/or related to the offset of the stimulus. In order to check this, we analyzed the aborted trials too, where the animal had broken the fixation. In this case the stimulus disappeared immediately and the animal got no reward. While the animal has broken the fixation the appearing eye movements could also elicit changes in the neuronal activity. In order to exclude the effects of eye movement- related activity in this case, the correlation between the interspike intervals and the normalized amplitude of the eye movements recorded during the experiment was computed. Fig 5D denotes the result of the linear regression analysis. This clearly shows the lack of connection (ρ^2^: 0.02, p>0.05) and in this way the absence of eye movement- related activity. Whether the change in activity is reward-related can be told by a simple examination of the PSTHs in relation to the offset of the stimulus: if the response is reward-related, no peak in the PSTH can be observed. If there is a peak in these histograms, the activity is likely to be related to the stimulus offset. Fig 5 shows the activity of a PAN, which responded with increased discharges to the offset of the stimulus.

### Response Characteristics of the High-Firing Neurons

**Fig 6:**
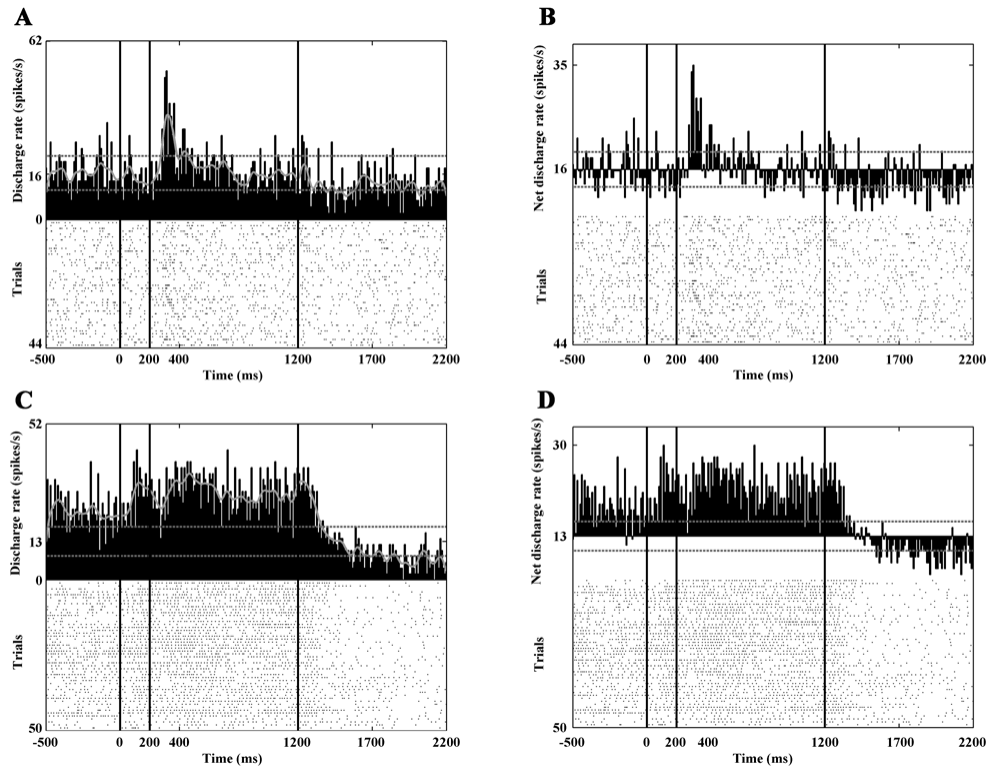
Marked responses to optic flow stimulation. Panels A and B show the activity of a HFN with a characteristic phasic response to the onset of the optic flow. Panels C and D show the responses of another HFN to static as well as to optic flow stimulation. Note the marked responses of these units to optic flow stimulation. The conventions are the same as in Fig 3.

Eighty-eight high-firing neurons were analyzed. The mean spontaneous discharge rate was 14.45 spikes/sec (SD: ±6.81). During the static phase 18 neurons increased and 18 neurons decreased their activity significantly (Fig 3C and 3D). Thirty-seven high-firing CN neurons responded to the optic flow stimulus. During the center-out optic flow 16 neurons and during center-in optic flow 20 neurons exhibited increased activity. The visual responses of this group are much clearer and stronger than those of the PANs and TANs (Table 2). Fig 4C and 4D and Fig 6 show the responses of three HFNs to optic flow (one cell with decreased and two cells with increased activity).

**Fig 7:**
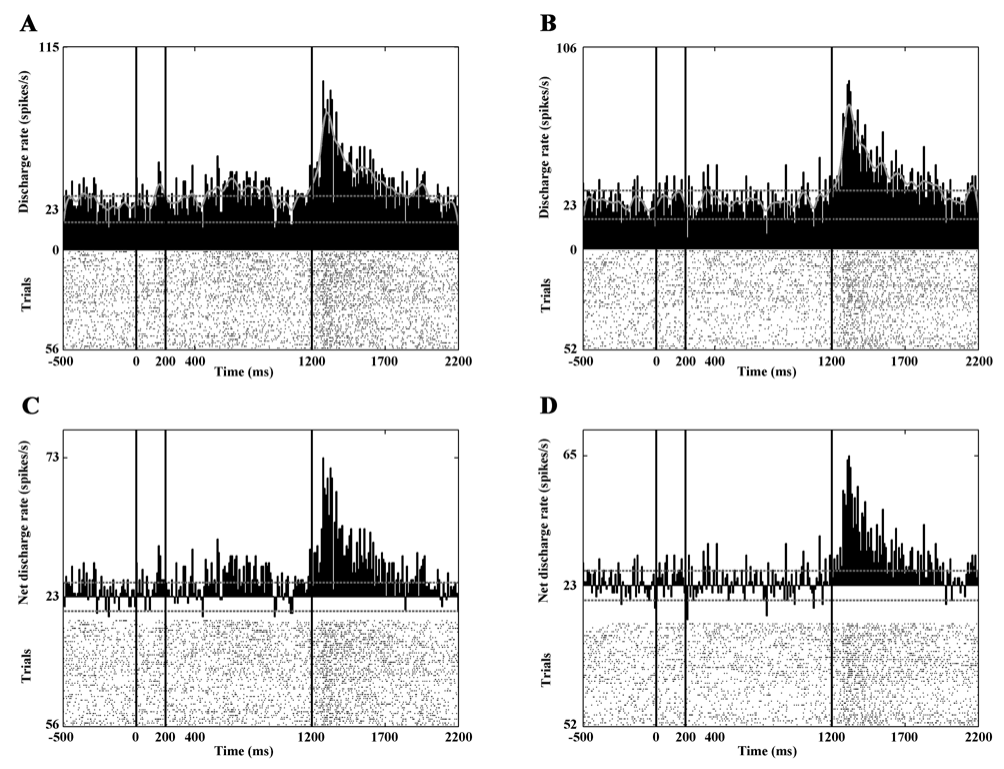
Selective responses to the direction of the optic flow. This figure demonstrates the activity of a HFN. Panels A (gross activity) and C (net activity) show the activities in response to center-out optic flow. Panels B (gross activity) and D (net activity) show the activities in response to center-in optic flow. Note that this neuron exhibits increased activity to center-out optic flow but no response to center-in optic flow. In other words, the neuron is selectively sensitive to the center-out direction. A strong reward-related activity can also be observed. The conventions are the same as in Fig 3.

During the reward phase, 30 neurons increased their activity and none of the high-firing neurons showed significantly decreased activity (Fig 3C and 3D and Fig 7). It was also found that altogether eight of the analyzed HFNs were active (seven of them with increased activity) during the offset of the stimulus. Similarly to the PAN, which was sensitive to the offset of the stimulus the HFNs with offset-related responses showed no eye movement connected activity.

### Response Characteristics of the Tonically Firing Neurons

Beside the PANs and HFNs a small number of CN neurons (28) were classified as tonically firing (TAN). The mean spontaneous discharge rate was 5.24 spikes/sec (SD: ± 2.37). During the static stimulation phase 11 neurons showed significantly increased and only 2 decreased activity. Seven of them responded by increasing their discharge rate to the optic flow stimulus (3 to the center-out and 5 to the center-in stimulus). The visual responses of the TANs were moderate or weak (Table 2). During the reward phase 6 neurons increased their activity. The offset of the stimulus influenced the activity of only one TAN. Similarly to the PAN and the HFNs, which were sensitive to the offset of the stimulus the TAN with offset-related response showed no eye movement connected activity.

### Sensitivity to the Direction of the Optic Flow

Altogether 74 (30 PANs, 36 HFNs and 8 TAN) of the 346 analyzed CN neurons showed significant activity change upon optic flow stimulation. In the majority of the analyzed neurons this change was not direction-dependent. Direction-dependent activity change was observed in twenty neurons (9 PAN, 8 HFN, 3 TAN). About half of the selective neurons (11 neurons) responded stronger to center-in stimulus while the second half of them (9 neurons) responded stronger to the center out optic flow. Fig 7 shows the PSTHs of a direction sensitive HFN.

At the population level there was no significant difference between the proportions of direction preferences within PANs, HFNs and TANs (χ-square test; χ^2^(2) = 1.001, df = 2, level of significance: 0.05). Thus, the PANs, the HFNs and the TANs of the CN appear to code the direction of the optic flow to a similar extent.

### Activity of the Neuron Groups during the Different Phases of the Behavioral Paradigm

Similarly to other studies [37, 38], we found using one-way ANOVA and the following post hoc analysis that the background activity of the HFNs was significantly higher (p<0.001) than that of the PANs and the TANs (Table 2). To exclude the effect of the background activities, we subtracted these from the gross activities and so calculated the net firing rates. For the further analyses, the absolute values of the net discharge rates were applied. Table 2 provides detailed information about the net discharge rates of the PANs, HFNs and TANs by phase (i.e. static, dynamic, reward). A one-way ANOVA indicated significant variance (p<0.01). The subsequent post-hoc analysis (Tukey's HSD) revealed that the HFNs were both the most sensitive and exhibited the most vigorous responses throughout all the phases of the fixation paradigm. In summary, the activity of the HFNs differed significantly from the activity of both PANs and TANs, while the activities of the latter two did not differ significantly.

## Discussion

In the present study we managed to provide a detailed description of the visual response profile of different CN neurons in the brain of behaving cats, after having classified them according to their electrophysiological characteristics. To our knowledge, such observations have not been made before.

In the last few decades, behaving animal models have gradually gathered ground in neurophysiology, due to their advantages over anesthetized, paralyzed models. Behaving animals can be relatively easily used after brief behavioral training for several experimental purposes, but this is unfortunately not the case in visual and multisensory electrophysiological research. In visual electrophysiology, the investigated neurons often exhibit visuomotor activity (e.g. activity changes during saccades). This makes a continuous monitoring of eye movements indispensable. To minimize the influence of eye movements on the neuronal activities, the animal had to maintain fixation during the whole length of the trials. In this way the eye movement- related components of the activity can be excluded. During the analysis of neuronal responses, which were correlated to stimulus offset (see in Results) the aborted trials were also analyzed where the animal has broken the fixation. In these cases because of the lack of fixation the effects of eye movements on the neuronal activities were also investigated. The correlation between the interspike intervals and the normalized amplitude of the eye movements recorded during the experiments revealed no eye movement- correlated activity among the CN neurons, which were sensitive to the offset of the visual stimuli.

In a recent study we gave a detailed description a new feline model for chronic visual electrophysiological recordings [29]. This model yielded a relatively long recording time per day throughout several years from the same animal, with continuous eye movement control and stable head position. This model was also the basis of the present study.

Turning now to the discussion of the results, the most important achievement of this study may be the electrophysiological categorization of neurons in the feline CN. Similarly to earlier findings in rodents and primates [18, 19, 39–43] PANs, TANs and HFNs were found. The validity and applicability of this classification is signified by the fact that over 97% of the recorded CN units fell into one of the three major categories, and it was only 3% that did not fit any of them. Earlier studies suggested a strong correspondence between the three biggest anatomical (medium spiny, cholinergic and parvalbumin immunpositive GABAergic interneurons) and electrophysiological (PAN, TAN, HFN) groups of the CN neurons. A growing body of evidence suggests that PAN neurons correspond to the medium spiny projection neurons, HFNs to the parvalbumin immunpositive GABAergic interneurons and the TFNs to the cholinergic interneurons [21–28]. As for the uncategorizable neurons (three percent in this study), these can belong to one of the numerous interneuron types of the caudate nucleus, such as the neuropeptide Y-, nitric oxide synthase- and somatostatin- containing [44, 45], calretinin immunpositive [46], tyrosine hydroxylase immunpositive [26, 47], cholecystokinin immunpositive [48] and vasoactive intestinal polypeptide immunpositive [49–51] interneurons—with no claim to being exhaustive. The low prevalence of these interneurons in the feline brain is in line with earlier findings in rodents and primates where the prevalence of each group of these interneurons is under 1%.

A further finding that fits with earlier studies in other species is that the majority of the CN units in the feline brain appear to be PAN (64% in our sample). In other species 77–97% of the striatal projection neurons belong to this most probably GABAergic cluster. Striatal projection neurons comprise up to 97% of the rodent striatum, while this proportion is significantly lower in higher vertebrates, especially in primates [20, 46, 52–55]. It must be noted that PANs are often difficult to detect because of their extremely low background activity [21, 56], which means that the sixty-four percent finding may be an underestimation due to undersampling, which, in turn, can also have an effect on the estimations of the two other groups.

As for the two other major groups, our results suggest that they are much less prevalent than PAN (HFN 25% and TAN 8%). This is also in line with the results of earlier rodent and primate studies. The parvalbumin immunpositive GABAergic interneurons comprise roughly 1–20% of the striatal neurons of rats and primates [54], while the cholinergic group adds up to only about 10 percent of the CN neurons (up to 10% in primates [57], an 2% in cats [58]).

Beyond the electrophysiological classification of the neurons in the feline CN, we also determined the visual response characteristics of the neurons belonging to the three major classes. The applied dynamic stimulus was quite new in this context, as hitherto optic flow has not been applied to investigate CN neurons. In our earlier studies we demonstrated that the CN neurons are strongly sensitive to very low spatial and high temporal frequency sinewave gratings and exhibit narrow temporal and spatial frequency tuning [11, 12]. It has been hypothesized for some time that neurons with such spatio-temporal visual response characteristics could have a role in the processing of optic flow [13, 14]. In the present study we revisited this hypothesis and provided the first piece of direct evidence on the processing of optic flow in the feline CN.

After the categorization of the CN units based on their electrophysiological properties, it was possible to investigate their visual response characteristics by group. Our results demonstrate that both the static and the dynamic components of the visual information are represented in the CN. The PANs and TANs were more sensitive to static than to dynamic visual stimulation, that is, they responded to the random dot patterns, but not to the optic flow. On the other hand, HFNs were almost equally sensitive to both static and dynamic stimulation (i.e. approximately the same proportion of these neurons responded to random dot pattern and to optic flow stimulation). The stimulus offset modulated the activity of a significant proportion of HFNs, which was not observed with PANs and TANs. This suggests that the PANs and TANs are primarily sensitive to static, continuous, unchanging visual stimuli. As mentioned before, the response characteristics of the HFNs are different: these neurons seem to be equally responsive to both static and dynamic stimulation (including stimulus offset). Furthermore, the net activity changes of the HFNs were significantly stronger than what was observed in PANs and TANs, regardless of the actual phase of the behavioral paradigm. These suggest that HFNs are the most sensitive units in the CN to visual stimuli.

We have also investigated whether the direction of the optic flow (center-in or center-out) is reflected in the activity of the CN neurons. The majority of the CN units showed no such sensitivity. Of the other hand a smaller population of the CN neurons were sensitive to this aspect of the stimulation. About half of the direction sensitive units responded stronger to center in stimuli and the second half of them were more sensitive to center out flow field. The sensitivity of the CN neurons to optic flow gives further support to the hypothesis that the CN neurons participate in motion perception, most probably in the perception of changes in the visual environment during self-motion [12–14].

In summary, we consider the following as the most important achievements of this study:

First, we managed to utilize our head- restrained, eye movement- controlled behaving feline model [29] in a visual electrophysiological study aimed at the analysis and classification of CN neurons in terms of their visual responsiveness.

Second, we described that different CN neuronal groups (PAN, TAN, HFN) are differently sensitive to static and dynamic visual stimulation. PAN and TAN neurons are primarily sensitive to static stimuli, while HFNs are primarily sensitive to changes in the visual environment of the animal. By this we also showed that visually sensitive neurons in the feline CN can be classified similarly to what had previously been found in other species.

Third, we managed to demonstrate optic flow processing in the feline CN, which emphasizes the role of this structure in the detection and processing of visual information related to motion.
